# Secondary symbionts affect aphid fitness and the titer of primary symbiont

**DOI:** 10.3389/fpls.2023.1096750

**Published:** 2023-01-27

**Authors:** Shen Liu, Xiaobei Liu, Tiantao Zhang, Shuxiong Bai, Kanglai He, Yongjun Zhang, Frédéric Francis, Zhenying Wang

**Affiliations:** ^1^ State Key Laboratory for Biology of Plant Diseases and Insect Pests, Institute of Plant Protection, Chinese Academy of Agricultural Sciences, Beijing, China; ^2^ Functional and Evolutionary Entomology, Gembloux Agro-Bio Tech, University of Liège, Gembloux, Belgium

**Keywords:** Hamiltonella defensa, Regiella insecticola, Rhopalosiphum maidis, aphid fitness, symbiont titer

## Abstract

Bacterial symbionts associated with aphids are important for their ecological fitness. The corn leaf aphid, *Rhopalosiphum maidis* (Fitch), is one of the most damaging aphid pests on maize and has been reported to harbor *Hamiltonella defensa* and *Regiella insecticola* while the effects of the secondary symbionts (S-symbionts) on host ecology and primary symbiont *Buchnera aphidicola* remain unclear. Here, four aphid strains were established, two of which were collected from Langfang - Hebei Province, China, with similar symbiont pattern except for the presence of *H. defensa*. Two other aphid strains were collected from Nanning - Guangxi Province, China, with the same symbiont infection except for the presence of *R. insecticola*. Phylogenetic analysis and aphid genotyping indicated that the S-symbiont-infected and free aphid strains from the same location had identical genetic backgrounds. Aphid fitness measurement showed that aphid strain infected with *H. defensa* performed shortened developmental duration for 1^st^ instar and total nymph stages, reduced aphid survival rate, offspring, and longevity. While the developmental duration of H-infected strains was accelerated, and the adult weight was significantly higher compared to the H-free strain. Infection with *R. insecticola* did not affect the aphid’s entire nymph stage duration and survival rate. As the H-strain does, aphids infected with *R. insecticola* also underwent a drop in offspring, along with marginally lower longevity. Unlike the H-infected strain, the R-infected strain performed delayed developmental duration and lower adult weight. The *B. aphidicola* titers of the H-infected strains showed a steep drop during the aphid 1^st^ to 3^rd^ instar stages, while the augmentation of *B. aphidicola* titers was found in the R-infected strain during the aphid 1^st^ to 3^rd^ instar. Our study investigated for the first time the effect of the S-symbionts on the ecology fitness and primary symbiont in *R. maidis*, indicating that infection with secondary symbionts leads to the modulation of aphid primary symbiont abundance, together inducing significant fitness costs on aphids with further impact on environmental adaptation and trophic interactions.

## Introduction

The close relationship between insects and symbionts is widespread (Moran et al., 2008), and infection with endosymbionts can be a key innovation that brings diversification to aphid ecology and fitness (Douglas, 2015). Aphids depend on the indispensable primary symbiont *Buchnera aphidicola* to provide nutritional supplementation that is lacking in their diet (Chong et al., 2019). Also, *B. aphidicola* may confer heat tolerance to aphids (Zhang et al., 2019), and the fluctuating number of endosymbiont cells could contribute to the adaptation of aphids to their environment (Neiers et al., 2021), consequently helping aphids to have a better adaption to temperatures or environmental variations.

In addition to *B. aphidicola*, other symbionts are not essential for aphids’ survival or reproduction but provide essential services for their hosts, which are referred to as secondary symbionts (S-symbionts). To date, nine known S-symbiont have been detected in aphids, namely *Arsenophonus*, Fukatsuia symbiotica, *Hamiltonella defensa*, *Regiella insecticola*, *Rickettsia*, *Rickettsiella*, *Serratia symbiotica*, *Spiroplasma* & *Wolbachia* (Guo et al., 2019; Patel et al., 2019). More functions of S-symbiont are being explored. S-symbionts may confer aphid protection against parasitoids (Oliver and Higashi, 2019), influence the interactions between aphids and their predators (Tsuchida et al., 2010), reduce the entomopathogen fungal infection on aphid bodies (Scarborough et al., 2005), help aphids mediate the plant defense responses (Li et al., 2019), improve the aphid susceptibility to insecticides (Skaljac et al., 2018), and enhance the aphid tolerance to heat (Montllor et al., 2002).

Aphids display relationships with symbionts that confer fitness benefits or costs to themselves (Leybourne et al., 2022). *H. defensa* has been well-studied in *Acyrthosiphon pisum* (Harris) (McLean and Godfray, 2015), illustrating S-symbiont could confer aphid protection against parasitoids while imposing life-history costs (Cayetano et al., 2015) and may exhibit a detrimental effect on aphid fitness with a 60% fecundity reduction on average (Simon et al., 2011). However, the positive effect of *H. defensa* on aphid fecundity has also been reported (Łukasik et al., 2013), such as leading to an increase in adult weight of *Sitobion miscanthi* (Takahashi) (Li et al., 2018). *R. insecticola* has also been demonstrated for its ability to protect aphids against parasitoids (Vorburger et al., 2010). Aphids infected with *R. insecticola* showed an enhanced ability to reproduce under parasitoid pressure (Luo et al., 2020b). Moreover, *R. insecticola* can improve the plasticity of aphid nymph development and fecundity in plant-insect interactions (Wang et al., 2016). Also, *R. insecticola* may inhibit the production of winged aphids while its negative effects on aphids were environmentally dependent (Liu et al., 2019).

S-symbionts may influence the abundance of the *B. aphidicola* positively or negatively (Laughton et al., 2014), sometimes these impacts depend on aphid genotypes or symbiont strains. In *A. pisum*, infection with *H. defensa* correlated with decreased *B. aphidicola* titer in the aphid strains AS3 and ZA17 but increased *B. aphidicola* titer in the aphid strain WA4 (Martinez et al., 2014). Meanwhile, infection with *H. defensa* may indirectly improve the fitness of aphids by stimulating the abundance of *B. aphidicola* (Li et al., 2018). However, infection with *Rickettsiella* induced a strong reduction in *B. aphidicola* titer (Leclair et al., 2017). Also, some S-symbionts may form a co-obligatory symbiosis with *B. aphidicola* to jointly supply essential nutrients to aphids (De Clerck et al., 2015; Meseguer et al., 2017). Therefore, symbiont presence and titer may be closely related to aphids’ fitness.

The corn leaf aphid, *Rhopalosiphum maidis* (Fitch), is one of the most economically damaging aphid pests on maize (*Zea mays*) and can transmit several damaging maize viruses, resulting in serious yield losses (Chen et al., 2019; Chen et al., 2020), whereas only sporadic reports focus on *R. maidis* symbionts. Previous studies have tested the symbiont combination of *R. maidis* and other aphid species collected across Morocco and found that aphid symbiont combinations were mainly host-specific (Fakhour et al., 2018), detected the infection patterns of seven facultative symbionts of *R. maidis* distributed in 37 geographical populations in China (Guo et al., 2019), and demonstrated that some symbionts may have a direct effect on aphids’ adaptation to different maize management systems (Csorba et al., 2022). Nevertheless, there is no report on the impact of *H. defensa* and *R. insecticola* on *R. maidis* ecology and *B. aphidicola* abundance.

To address these important deficiencies, S-symbionts-infected (*H. defensa* or *R. insecticola*) and free aphid strains were established with similar genetic backgrounds to evaluate the impacts of S-symbionts on aphids’ fitness and *B. aphidicola* titers. Nymph durations and survival time were recorded as aphids aged, aphid fitness indices were measured, and all three symbiont titers were measured by qPCR in each aphid developmental stage. Our study aims to investigate the potential trade-off that aphids could benefit from carrying S-symbionts while undergoing their own energy reallocation and how that could affect the *B. aphidicola* titer. Our findings contribute to a better understanding of symbiotic interactions in *R. maidis*.

## Material and methods

### Aphid strains and rearing

Four aphid strains were established initially from single aphids collected in different locations in China (**Table 1**). All aphid strains were maintained on barley seedlings (*Hordeum vulgare* L.) in the laboratory at a constant 25 ± 1°C with a 75% relative humidity and a 16 hours daily light cycle. To eliminate any adverse effects from host plant alteration, aphids were used for the following experiments after 5 generations. The symbiont pattern status of all aphid strains was periodically confirmed by PCR.

**Table 1:** Symbionts infection in different aphid strains.

Locality	Coordinates	Aphid strains	*Buchnera aphidicola*	*Hamiltonella defensa*	*Regiella insecticola*
Langfang City, Hebei Province	39°30’57.950”N;	H-free	+		
	116°36’53.396”E	H-infected	+	+	
Nanning City, Guangxi Province	22°49’39.400”N;	R-free	+		
	108°22’36.076”E	R-infected	+		+

“+” indicated infection with the endosymbiont.

### Aphid DNA extraction and endosymbionts detection

Aphid samples were collected from maize plants and total aphid genomic DNA was extracted using 1 mL 0.1M Tris-HCL buffer and 8 µl proteinase K as described by Myint et al. (2021) with minor modifications. One aphid was crushed in 30 µl volume buffer in the PCR tube and then centrifuged at 8,000 rpm for 1 min at RT, the homogenate was subsequently incubated at 65°C for 30 min, 25°C for 2 min, 96°C for 10 min, and final hold at 4°C.

All DNA samples were screened for the nine known S-symbionts mentioned above, with PCR using universal primers and specific primers based on symbionts’ 16S rRNA gene sequences. All primer sequences are listed in **Supplementary Material Table S1**. PCR cycling conditions were 94°C for 5 min followed by 35 cycles of 94°C for 30s, 56°C for 30 s, 72°C for 1 min, 72°C for 10 min for the final extension and hold at 10°C. The reaction products were analyzed with a model 3500 ABI PRISM DNA sequencer (Perkin-Elmer, New York, USA). The nucleotide sequences of the *H. defensa* and *R. insecticola* 16S rRNA partial genes of *R. maidis* described in this paper have been deposited in GenBank under accession numbers ON248614 and ON248615, respectively.

### Aphid microsatellite genotyping and phylogenetic analysis

**Figure 1:**
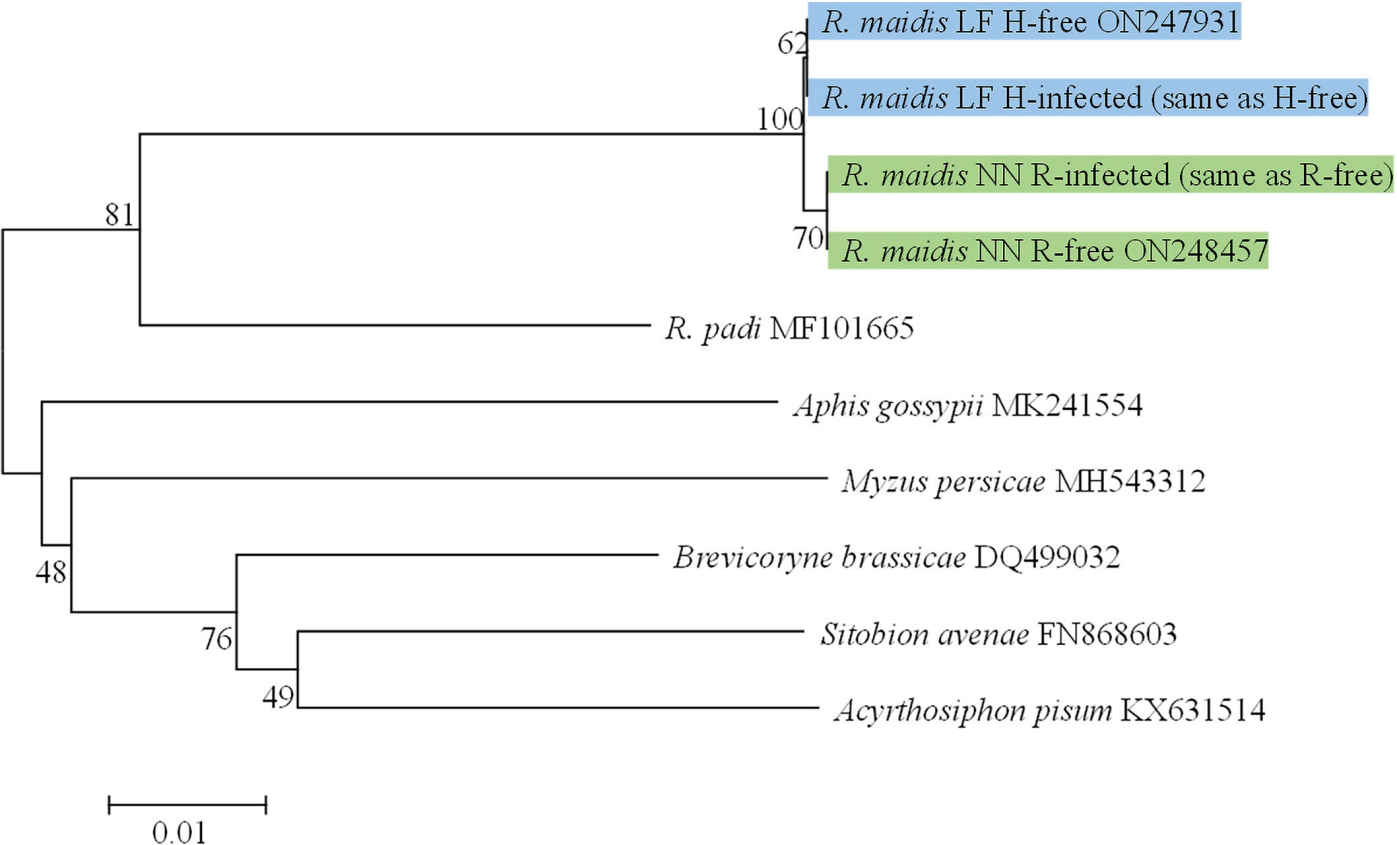
Phylogenetic trees of *COI* gene from different aphid strains based on neighbor-joining (NJ) method. Green frames, aphid strains from Nanning City. Blue frames, aphid strains from Langfang City.

To ensure the consistency of the geographic genetic background, the mitochondrial cytochrome oxidase I (*COI*) gene sequences of all aphid strains were amplified and were used to build a neighbor-joining tree with the Kimura 2-parameter model and 1000 bootstrap replications with MEGA 7.0.26 (Kumar et al., 2001). The *COI* gene nucleotide sequence of H-free, H-infected, R-free, and R-infected aphid strains described in this paper have been deposited in GenBank (**Figure 1**).

To eliminate the effect of aphid genotype on subsequent experiments, aphids were genotyped based on three microsatellite loci, *R3.171*, *R5.10*, and *S17b*, which were successfully isolated from *R. padis*, considering that no microsatellite loci have been isolated from *R. maidis* and these two aphids were related species belonging to the same genus *Rhopalosiphum* (Wilson et al., 2004; Leybourne et al., 2020a). Aphid genotypes were determined based on the pattern of PCR product sizes from the amplified microsatellite loci described in this paper, which gene sequences have been deposited in GenBank under accession number ON262199-ON262202 (**Table 2**). Microsatellite loci and *COI* gene primers are shown in **Table S1**. PCR cycling conditions were described above with different annealing temperatures.

**Table 2:** Genotype and allele sizes of different aphid strains.

Aphid strains	Collected City	Genotype assigned	Microsatellite marker allele sizes (bp)		
			*R3.171*	*R5.10*	*S17b*
H-free	LF	A	265	246, 247	152, 153
		B	265	246, 247	167-175
		C	265	255, 257	167-175
		D	265	255, 257	152, 153
H-infected	LF	A	265	246, 247	152, 153
		D	265	255, 257	152, 153
R-free	NN	E	265	256, 257	152, 153
		F	265	256, 257	173-178
		G	265	256, 257	159, 160
		H	267	243	159, 160
R-infected	NN	E	265	256, 257	152, 153
		G	265	256, 257	159, 160

### Aphid fitness measurement

Adult aphids from different strains were selected and placed individually in Petri dishes containing barley seedlings wrapped with wet cotton and placed in an incubator with the same condition as aphid rearing. After a period of time, the adult aphid and the redundant newborn nymphs were removed from each Petri dish with only one newborn nymph left. These nymphs were allowed to develop into adults until they completed their entire lifecycle, and fresh barley seedlings were replaced every 5 days.

Aphid nymph instar durations were recorded at half-day intervals and monitored by tracking molting. Aphid fitness indices were measured with at least 30 aphids for each strain, including developmental time (days from birth to first reproduction), the total number of offspring, and longevity. The weight of 20 newly matured adults was recorded which performed at least 16 replicates and no alate aphid was observed during the entire aphid lifecycle.

### Real-Time qPCR of the aphid symbiont titers

The population sizes of symbionts were quantified by the ratio of the copy number of the symbionts’ 16S rRNA gene to that of the *ef1α* gene to determine whether the presence of S-symbionts influence the relative abundance of *B. aphidicola*. DNA was extracted from different developmental stages of all the aphid strains, for each aphid stage, 10 aphids were sampled as a biological replicate and 3 biological replicates were performed, and no less than 4 technical replicates were performed for each biological replicate.

Quantitative PCR (qPCR) was performed by ABI Prism 7,500 Fast Real-Time PCR System (Thermo Fisher Scientific, Waltham, MA, USA) using specific primers provided in **Table S1**, which were designed in this study according to each symbiont 16S rRNA gene, with amplification efficiency 103.8, 107.8, 107.7, and 106.0% for *B. aphidicola*, *H. defensa*, *R. insecticola*, and *ef1α* gene, respectively. The qPCR reaction volume was 20 µl volumes containing 10 µl of 2×*PerfectStart* ^®^ Green qPCR Mix (Trans, Beijing, China), 0.4 µl of Passive Reference Dye II (50×), 0.4 µl of each primer, 1 µl of DNA, and 7.8 µl Nuclease-free Water. Cycling conditions were 95°C for 30 s, then 40 cycles of 95°C for 5 s and 60°C for 30 s. 4 technical replicates were performed for each sample.

Standard curves were established using serial dilutions of plasmid DNA containing different target genes, which covers the range from 10^3^ to 10^9^ copies, where the x-axis is the log of plasmid DNA concentration and the y-axis is the Ct value, while the gene copy numbers were calculated using the method as described in (Whelan et al., 2003).

### Statistical analyses

All the statistical analyses were performed with IBM SPSS Statistics software (ver. 26.0, SPSS Inc.). The survival time of each aphid strain was visualized as Kaplan-Meier survival curves and was assessed with the Log-rank (Mantel-Cox) test. The aphid nymph instar durations and fitness indices of different aphid strains were compared by the Student’s *t*-test.

## Results

### Establishment of aphid strains

All aphid strains harbored the primary symbiont *B. aphidicola*. Four aphid strains were established, compromising *H. defensa*-free (H-free) and *H. defensa*-infected (H-infected) from Langfang, *R. insecticola*-free (R-free) and *R. insecticola*-infected (R-infected) from Nanning (**Table 1**).

### Phylogenetic analysis and aphid genotype

Phylogenetic analysis indicated that the *COI* gene sequences of H-free and H-infected aphid strains were strictly identical but distinct from the other two aphid strains (R-free and R-infected) which were strictly identical (**Figure 1**). In addition, based on the banding patterns of the 3 microsatellite PCR products, all aphid strains were grouped into one of eight genotypes (labeled A-H, **Table 2**). *H. defensa* and *R. insecticola* were detected in genotypes A and D, E and G, respectively, and the same genotypes were observed in S-symbiont-free aphid strains collected from the same location. Above all, S-symbiont-infected aphid strains and their equivalent S-symbiont-free aphid strains were from the same location, and have identical genetic backgrounds.

There was only one base pair difference in the *R5.10* microsatellite loci when comparing genotypes D and E. Based on this result, we selected these two genotypes and their corresponding four aphid strains to minimize the effect of the aphid genotype on aphid fitness and *B. aphidicola* titers.

### Effects of S-symbionts on aphid fitness

**Figure 2:**
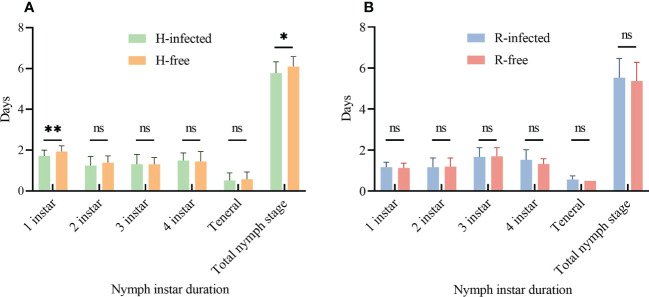
Aphid nymph instar duration of different aphid strains. **(A)** Nymph instar duration of H-infected and H-free aphid strains. **(B)** Nymph instar duration of R-infected and R-free aphid strains. The asterisk indicates significant differences based on the *t*-test for two-sample comparison: *P<0.05, **P<0.01, and ns nonsignificant.

Aphid demographic parameters were compared between S-symbiont infected strains and their corresponding S-symbiont free strains. To the aphid nymph instar duration, there was no significant difference when comparing the 2^nd^-4^th^ nymph stages of H-infected and H-free strains. Aphid strain infected with *H. defensa* had a shorter 1^st^ instar stage (1.7 d) and the total nymph stage (5.8 d) than that of the H-free strain (1.9 d, *t*=3.22, *P*=0.002; 6.1 d, *t*=2.50, *P*=0.015; **Figure 2A**). However, no significant difference was seen in the entire nymph stages between the R-infected and R-free strains (**Figure 2B**).

**Figure 3:**
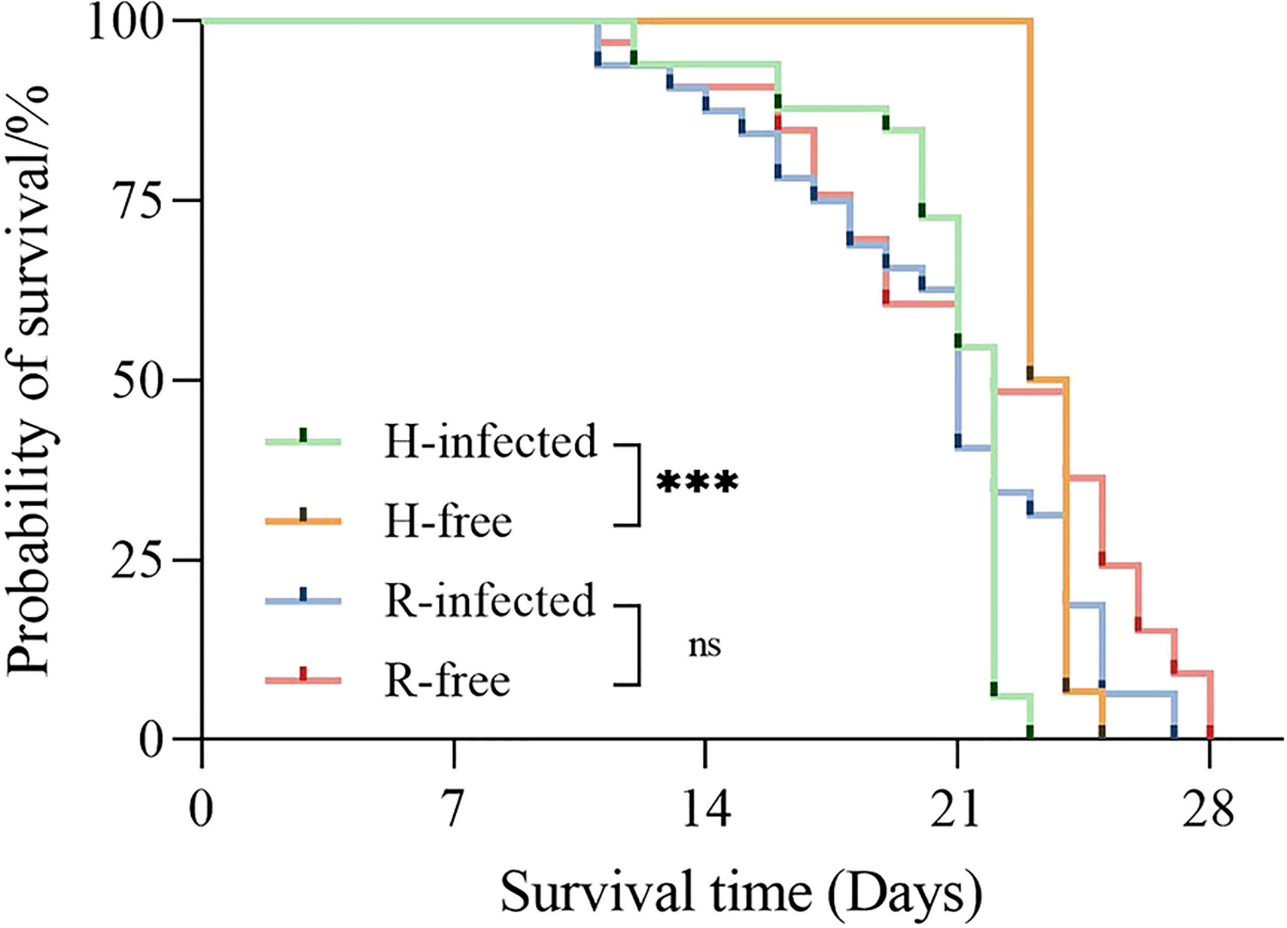
Survival curves of different aphid strains. The asterisk indicates significant differences based on the Log-rank (Mantel-Cox) test, ***P<0.001, and ns nonsignificant.

The survival time of the H-infected strain was much lower than the H-free strain (*P*<0.001), suggesting that aphid survival time was observably influenced by harboring *H. defensa.* However, no difference in survival time was observed between the R-free and R-infected strains (**Figure 3**).

**Figure 4:**
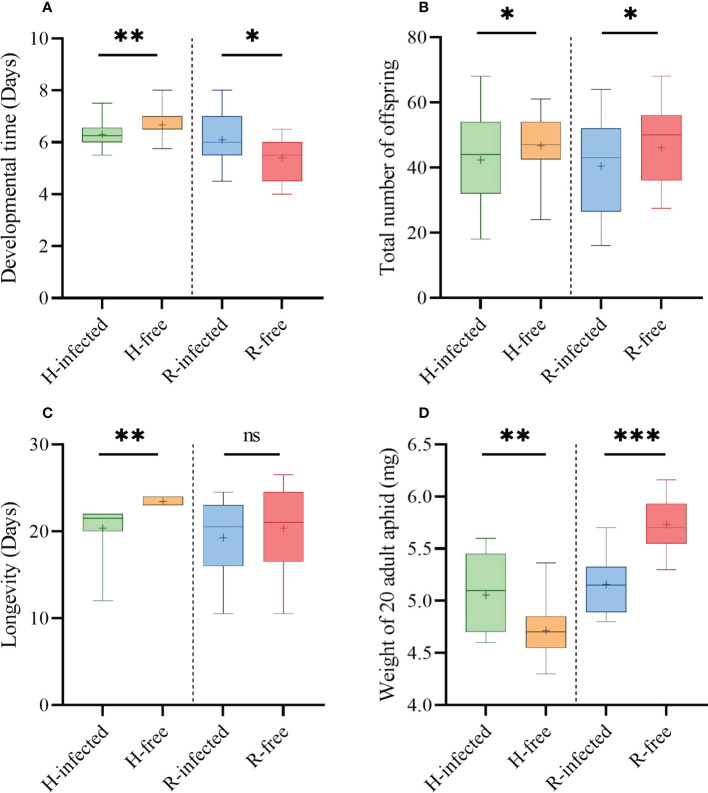
Fitness indices of different aphid strains. **(A)** Aphid developmental time. **(B)** Aphid total number of offspring. **(C)** Aphid longevity. **(D)** Weight of 20 newly matured aphid adults. Box plots: boxes, interquartile range (IQR); whiskers, minimum and maximum values; lines inside the boxes, median value; cross, mean value. The asterisk indicates significant differences based on the *t*-test for two-sample comparison: *P<0.05, **P<0.01, ***P<0.001, and ns nonsignificant.

The presence of S-symbiont had distinct influences on aphid fitness indices. The developmental time of H-infected strains (6.3 d) was accelerated compared with the H-free strains (6.7 d, *t*=3.19, *P*=0.002, **Figure 4A**). H-infected strains produced fewer offspring (44.0) and had shorter longevity (20.6 d) than the H-free strains (48.8, *t*=2.19, *P*=0.032, **Figure 4B**; 23.6 d, *t*=6.12, *P*=0.001, **Figure 4C**). While the weight of the H-infected strain (5.06 mg) was observably heavier in contrast to the H-free strain (4.71 mg, *t*=-2.30, *P*=0.005, **Figure 4D**).

Aphids infected with *R. insecticola* resulted in a significant increase in the developmental time (6.1 d) compared to the R-free strain (5.4 d, *t*=-2.29, *P*=0.030, **Figure 4A**). The fecundity of the R-infected strain (41.6) was significantly fewer than the R-free strain (48.0, *t*=2.02, *P*=0.047, **Figure 4B**), however, no significant difference was observed in longevity between the two aphid strains (**Figure 4C**). Furthermore, the weight of the R-infected strain (5.16 mg) differed sharply relative to that of the R-free strain (5.73 mg, *t*=-4.86, *P*=0.030, **Figure 4D**).

### Effect of S-symbionts on *B. aphidicola* titers

**Figure 5:**
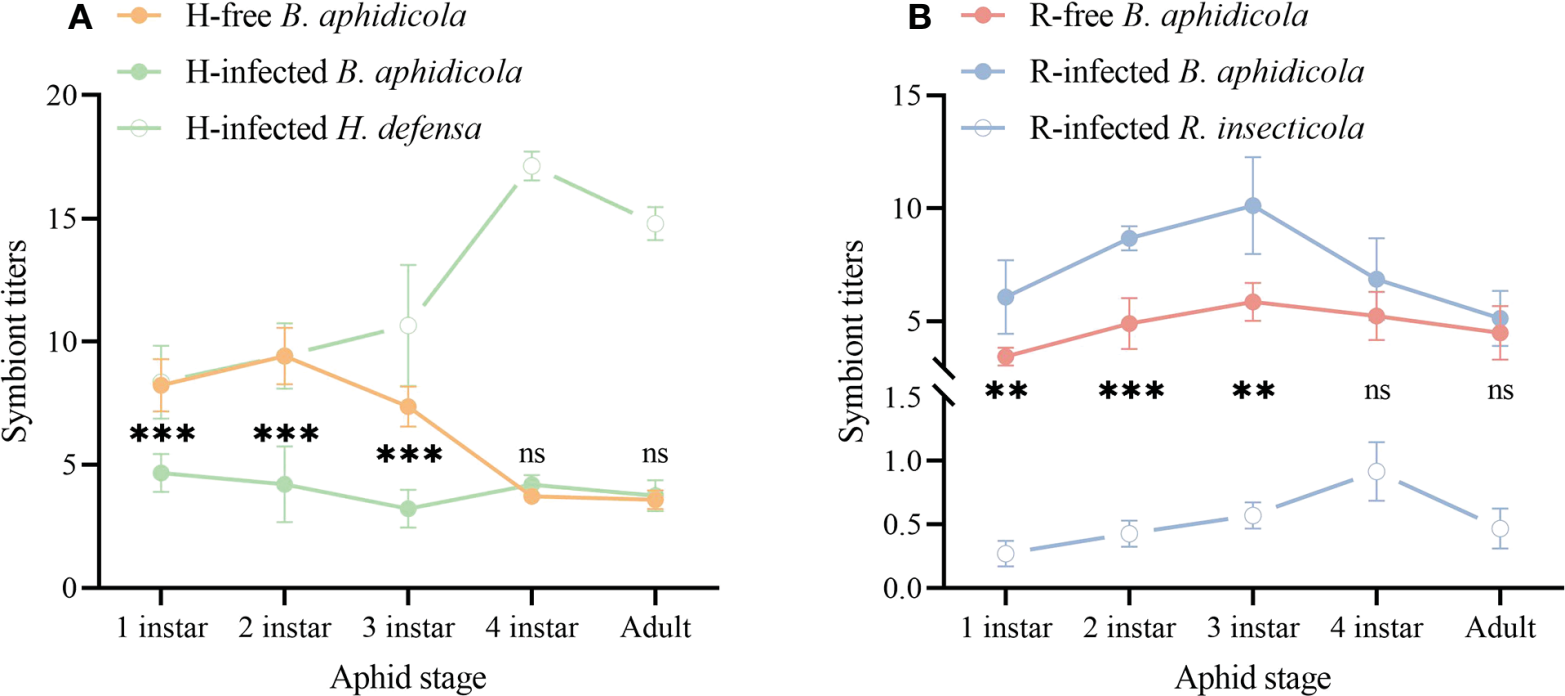
Symbiont titers of different aphid strains. **(A)** *B. aphidicola* and *H. defensa* titers. **(B)** *B. aphidicola* and *R. insecticola* titers. The asterisk indicates significant differences in *B aphidicola* titers between the S-symbiont-infected aphid strains and its equivalent S-symbiont-free aphid strains, based on the *t*-test for two-sample comparison: **P<0.01, ***P<0.001, and ns nonsignificant.

To address whether the infection of S-symbionts affects the titers of the aphid primary symbiont *B. aphidicola*, we measured the symbiont titers of all the aphid stages in each aphid strain. Both the S-symbionts titers showed a semblable variation tendency of falling after rising and had the highest value at the aphid 4^th^ instar, although the titer values of each differed 17.6 times (**Figures 5A, B**).

*B. aphidicola* titers of the H-free strain peaked at the aphid 2^nd^ instar before declining rapidly at later development stages and were strongly higher than the H-infected strain during the 1^st^-3^rd^ nymph stages. In the H-infected strain, *B. aphidicola* titers fluctuated slightly during the entire aphid development stages (**Figure 5A**). As for the R-free strain, the titers of *B. aphidicola* rose at the very beginning, reached the highest value at aphid 3^rd^ instar then fell. The variation of *B. aphidicola* titers in the R-infected strain showed a similar pattern and was significantly higher relative to that of the R-free strain during the 1^st^-3^rd^ nymph stages (**Figure 5B**). These results demonstrated that the titers of *B. aphidicola* were affected by S-symbionts infection.

## Discussion

Infection with symbionts is widespread within aphids, the latter live in intimate association with symbionts which can influence aphid reproduction and growth (Perreau et al., 2021). To date, the function of S-symbionts in *R. maidis*, an important pest of maize worldwide, remains to be explored. Our study investigated the effects of S-symbionts (*H. defensa* and *R. insecticola*) on *R. maidis* fitness and primary symbiont abundance. The variation in aphid fitness associated with the presence of S-symbionts might be an indirect consequence of the fluctuation of *B. aphidicola* abundance. This demonstrated a potential trade-off whereby aphids could benefit from carrying S-symbionts while undergoing a reallocation of their own energy.

To more thoroughly investigate how S-symbiont affects aphids, four natural aphid strains were established in this study through symbionts screening, microsatellite genotyping, and phylogenetic analysis. Aphid genotype could be a key factor in aphid performance (Karley et al., 2017), such as aphids’ weight could be affected by different aphid genotypes (Leybourne et al., 2020a). Besides, aphid genotype could have an effect on symbionts’ growth and abundance (Mouton et al., 2007), which may result in different aphids’ performances. Therefore, it is important to obtain different aphid strains with the same or similar genotypes. In our study, aphid strains without S-symbiont belonged to four genotypes respectively, which fortunately included genotypes of aphid strains infected with S-symbiont, which laid a foundation for the subsequent study of the symbiont function.

The growth of the aphid nymph stage may be affected by the presence of S-symbionts. As this experiment showed, the duration of the 1^st^ instar and the total nymph stage of the H-infected strain performed reduced developmental time, whereas the entire nymph stages between the R-infected and R-free strains were semblable. It’s worth noting that empirical studies have demonstrated that S-symbionts could specifically increase the duration of aphid 3^rd^ instar during the growth of *S. avenae* (Luo et al., 2020b), and pertinent studies have found that the main augmentation of *B. aphidicola* occurs beginning in the aphid 3^rd^ instar in *A. pisum* (Simonet et al., 2016). There seems to be a linkage between the duration of the aphid nymph stage and the symbiont abundance, and subsequent studies are needed to clarify the correlation by detecting the symbiont abundance.

It is widely accepted that symbionts played a fundamental role in aphid evolution (Henry et al., 2015), and both the benefits that symbionts conferred upon aphids and the entailed costs of infection affect aphid development (Oliver et al., 2014). In the present study, by reducing the aphid survival rate, the infection of *H. defensa* incurred significant fitness costs on the aphid, including the reduction of offspring which is closely dependent on the aphid’s longevity and is also lower than the H-free strains. Similar results have been reported for *A. pisum* (Leclair et al., 2017). Interestingly, the developmental time of H-infected strains was accelerated and their adult weight was observably heavier in contrast to the H-free strain, as previously recorded (Li et al., 2018).

Studies have found that *R. insecticola* did not affect the aphid survival rate, even if two different strains were tested (Luo et al., 2020b). The same results were found in our study. As the H-strain does, aphids infected with *R. insecticola* also underwent a drop in offspring, along with marginally lower longevity observed for the R-infected strain. This again proves the intimate connection between aphid longevity and offspring as we mentioned above. Unlike the H-infected strain, we observed a delay in the developmental time of the R-infected strain. A similar phenomenon has been found in *A. pisum*, where the presence of *R. insecticola* caused a delay in aphid oviposition (Laughton et al., 2014). Besides, the R-infected strain performed poorly in weight, indicating a fitness cost resulting from *R. insecticola* infection.

Infection with symbionts may bring fitness costs to aphids as previously reported (Leybourne et al., 2020a; Luo et al., 2020a), although this effect may depend on aphid genotypes (Leclair et al., 2017), host plants (Leybourne et al., 2020b), S-symbiont strains (Luo et al., 2020b), and symbionts’ density (Simonet et al., 2016). Our results showed that infection with the protective symbionts *H. defensa* and *R. insecticola* could lead to a partial negative effect on aphid growth and development, even though endosymbionts are maintained in aphids over time. Nevertheless, these two S-symbionts have been shown to protect aphids from natural enemies (Wu et al., 2022), *R. insecticola* could protect *A. pisum* from the aphid-specific fungal entomopathogen *Zoophthora occidentalis* (Parker et al., 2013), and *H. defensa* could reduce aphid susceptibility to insecticides (Li et al., 2021). This phenomenon reflects a trade-off in the close aphid-symbiont relationship that aphids could benefit from harboring symbionts while suffering from it may lead to redistribution of aphid energy (Zytynska et al., 2021). In our study, infection of *H. defensa* elevated the aphid weight and facilitated the developmental time against the cost of the drop in longevity and offspring. Interestingly, results showed that infection of *R. insecticola* did not benefit the aphid growth, as it slightly lowered the aphid longevity, delayed the developmental time, and caused a decrease both in aphid offspring and weight. Further study should be conducted to investigate the benefits conferred upon aphids as costly as *R. insecticola*, such as its effect on aphid parasitoid resistance, so as to have a better understanding of the overall effect of the symbiont on aphids.

Aphid primary symbiont *B. aphidicola* has the ability to synthesize essential amino acids and other nutrients needed by the host, which is closely connected to the growth and reproduction of aphids (Shigenobu et al., 2000). In aphids, S-symbionts may influence the primary symbiont titer positively or negatively (Burke et al., 2010; Laughton et al., 2014), depending on a variety of factors, aphid species, aphid genotype, symbiont strains, and other conditions (Leclair et al., 2017). In this study, primary symbiont abundance displayed a fluctuation inflicted by the infection of S-symbionts, as *B. aphidicola* titers of the H-infected strains showed a steep drop during the aphid 1^st^ to 3^rd^ instar. Reduction in *B. aphidicola* abundance is often associated with detrimental effects on aphid fitness (Koga et al., 2003). This could explain that the fitness cost of the H-infected strain may partly be due to the reduction of the *B. aphidicola* abundance caused by the infection of *H. defensa*. In addition, the abundance of *H. defensa* was quite large which may also be another reason for the aphid fitness cost, as evidenced by that aphid strains with higher densities of symbionts tend to be associated with shorter longevity (Mathé-Hubert et al., 2019). Besides, high *Spiroplasma* densities have also been shown to curtail flies’ lifespans (Herren and Lemaitre, 2011).

In contrast to the variation of *B. aphidicola* titers in the H-infected strain, the augmentation of *B. aphidicola* titers was found in the R-infected strain during the aphid 1^st^ to 3^rd^ instar. Although the high abundance of *B. aphidicola* did not benefit the R-infected strain in aphid developmental time, offspring and weight, no difference in longevity and survival rate were observed compared to the R-free strain. These results may once again verify the trade-off in aphids infected with S-symbionts as we mentioned above. Therefore, we speculated that infection with S-symbionts represents an impact first on *B. aphidicola* with a consequent impact on aphid fitness.

In general, our study established *R. maidis* strains with and without S-symbionts (*H*. *defensa* and *R*. *insecticola*) which had identical genetic backgrounds through symbionts screening, microsatellite genotyping, and phylogenetic analysis. Our results found that infection with S-symbionts had obvious effects on aphid fitness and *B. aphidicola* titers which depend on S-symbionts species, and illustrated the trade-off is a key constituent of co-evolution between aphids and symbionts. Together, our study contributes to symbiont function research by revealing the effect of S-symbiont on aphid ecology and the correlation with symbiont titers. Pertinent studies have found that the prevalence of S-symbionts in aphids may be influenced by seasonal temperatures, host plants, parasitoids, and aphid species (Vorburger and Rouchet, 2016; Guidolin and Cônsoli, 2017; Pons et al., 2022). According to our rough statistics, the prevalence of *Hamiltonella defensa* or *Regiella insecticola* was 12.32% (17/138) and 14.40% (19/132) at the location where we collected them. What we should also take into consideration in our further study is that the low prevalence of these two S-symbionts may also be correlated with the benefits and cost of keeping them in aphids. Previous studies demonstrated the effect of aphid genotype on *B. aphidicola* titers was dependent on aphid host plants (Zhang et al., 2016) and observed the wide variation in *B. aphidicola* titers among aphid strains was attributable to host genotype (Vogel and Moran, 2011). Although we tried to minimize the differences in aphid genetic background and established aphid strains with the closest genotypes, the result of *B. aphidicola* titers determination showed that two S-symbiont-free aphid strains had different fluctuation patterns along aphid development stages (**Figures 5A, B**). This implied that we cannot attribute this distinction to aphid collecting locations or host plants since all the aphid strains were maintained under the same rearing condition in the lab for more than 15 generations before being used in experiments. Therefore, the changes in *B. aphidicola* titers’ fluctuation pattern in S-symbiont-infected aphid strains may not only be due to the presence of S-symbionts but also to the difference in aphid genotypes. To address both the effect of S-symbionts and aphid genotypes on *B. aphidicola* titers, aphid S-symbionts crossed infections by hemolymph injection is needed in the future, i.e., to infect the S-symbiont-free aphid strain from Langfang City with *R. insecticola* and the one from Nanning City with *H. defensa*. Besides, our study was confined to the effect of single S-symbionts on aphids, more research should be performed to explore the consequences of S-symbiont coinfection on aphid and symbiont titers. In addition, host plant species impact the density of aphid symbionts (Wilkinson et al., 2001), and symbionts play an important role in mediating the defense response of plant-insect interaction (Li et al., 2019). Follow-up studies should also be carried out to clarify the effect of S-symbionts on aphid feeding behavior on different host plants, plant defense reaction, and aphid parasitoids, thereby providing information on utilizing the symbionts for pest control.

## Data availability statement

The datasets presented in this study can be found in online repositories. The names of the repository/repositories and accession number(s) can be found in the article/**Supplementary Material**.

## Author contributions

SL, FF, and ZW conceptualized the study. TZ, SB, KH, and YZ assisted in the experimental methods. SL performed the experiment, XL assisted in the experiment of aphid fitness measurement. SL wrote the manuscript. FF and ZW reviewed and amended the manuscript. ZW provided financial support. All authors contributed to the article and approved the submitted version.

## References

[B1] Burke G. Fiehn O. Moran N. A. (2010). Effects of facultative symbionts and heat stress on the metabolome of pea aphids. ISME. J. 4, 242–252. doi: 10.1038/ismej.2009.114 19907504

[B2] Cayetano L. Rothacher L. Simon J. C. Vorburger C. (2015). Cheaper is not always worse: strongly protective isolates of a defensive symbiont are less costly to the aphid host. Proc. Biol. Sci. 282, 20142333. doi: 10.1098/rspb.2014.2333 25473015 PMC4286048

[B3] Chen Y. Quan Y. Verheggen F. Wang Z. Francis F. He K. (2020). Differential thermal tolerance across life stages under extreme high temperatures crossed with feeding status in corn leaf aphid. Ecol. Entomol. 46, 533–540. doi: 10.1111/een.12998

[B4] Chen W. Shakir S. Bigham M. Richter A. Fei Z. Jander G. (2019). Genome sequence of the corn leaf aphid (*Rhopalosiphum maidis* Fitch). Gigascience 8, giz033. doi: 10.1093/gigascience/giz033 30953568 PMC6451198

[B5] Chong R. A. Park H. Moran N. A. (2019). Genome evolution of the obligate endosymbiont *Buchnera aphidicola* . Mol. Biol. Evol. 36, 1481–1489. doi: 10.1093/molbev/msz082 30989224

[B6] Csorba A. B. Fora C. G. Bálint J. Felföldi T. Szabó A. Máthé I.. (2022). Endosymbiotic bacterial diversity of corn leaf aphid, *Rhopalosiphum maidis* Fitch (Hemiptera: Aphididae) associated with maize management systems. Microorganisms 10, 939. doi: 10.3390/microorganisms10050939 35630383 PMC9145372

[B7] De Clerck C. Fujiwara A. Joncour P. Léonard S. Félix M. L. Francis F.. (2015). A metagenomic approach from aphid’s hemolymph sheds light on the potential roles of co-existing endosymbionts. Microbiome 3, 63. doi: 10.1186/s40168-015-0130-5 26667400 PMC4678535

[B8] Douglas A. E. (2015). Multiorganismal insects: diversity and function of resident microorganisms. Annu. Rev. Entomol. 60, 17–34. doi: 10.1146/annurev-ento-010814-020822 25341109 PMC4465791

[B9] Fakhour S. Ambroise J. Renoz F. Foray V. Gala J. L. Hance T. (2018). A large-scale field study of bacterial communities in cereal aphid populations across Morocco. FEMS. Microbiol. Ecol. 94, fiy003. doi: 10.1093/femsec/fiy003 29346623

[B10] Guidolin A. S. Cônsoli F. L. (2017). Symbiont diversity of aphis (Toxoptera) citricidus (Hemiptera: Aphididae) as influenced by host plants. Microb. Ecol. 73, 201–210. doi: 10.1007/s00248-016-0892-8 27872949

[B11] Guo J. Liu X. Poncelet N. He K. Francis F. Wang Z. (2019). Detection and geographic distribution of seven facultative endosymbionts in two *Rhopalosiphum* aphid species. MicrobiologyOpen 8, e00817. doi: 10.1002/mbo3.817 30912316 PMC6692527

[B12] Henry L. M. Maiden M. C. Ferrari J. Godfray H. C. (2015). Insect life history and the evolution of bacterial mutualism. Ecol. Lett. 18, 516–525. doi: 10.1111/ele.12425 25868533

[B13] Herren J. K. Lemaitre B. (2011). *Spiroplasma* and host immunity: activation of humoral immune responses increases endosymbiont load and susceptibility to certain gram-negative bacterial pathogens in *Drosophila melanogaster* . Cell Microbiol. 13, 1385–1396. doi: 10.1111/j.1462-5822.2011.01627.x 21740495

[B14] Karley A. J. Emslie-Smith M. Bennett A. E. (2017). Potato aphid *Macrosiphum euphorbiae* performance is determined by aphid genotype and not mycorrhizal fungi or water availability. Insect Sci. 24, 1015–1024. doi: 10.1111/1744-7917.12445 28213920

[B15] Koga R. Tsuchida T. Fukatsu T. (2003). Changing partners in an obligate symbiosis: a facultative endosymbiont can compensate for loss of the essential endosymbiont *Buchnera* in an aphid. Proc. Biol. Sci. 270, 2543–2550. doi: 10.1098/rspb.2003.2537 14728775 PMC1691542

[B16] Kumar S. Tamura K. Jakobsen I. B. Nei M. (2001). MEGA2: molecular evolutionary genetics analysis software. Bioinformatics 17, 1244–1245. doi: 10.1093/bioinformatics/17.12.1244 11751241

[B17] Laughton A. M. Fan M. H. Gerardo N. M. (2014). The combined effects of bacterial symbionts and aging on life history traits in the pea aphid, *Acyrthosiphon pisum* . Appl. Environ. Microbiol. 80, 470–477. doi: 10.1128/AEM.02657-13 24185857 PMC3911086

[B18] Leclair M. Polin S. Jousseaume T. Simon J. C. Sugio A. Morlière S.. (2017). Consequences of coinfection with protective symbionts on the host phenotype and symbiont titres in the pea aphid system. Insect Sci. 24, 798–808. doi: 10.1111/1744-7917.12380 27514019

[B19] Leybourne D. J. Bos J. I. B. Valentine T. A. Karley A. J. (2020a). The price of protection: a defensive endosymbiont impairs nymph growth in the bird cherry-oat aphid, *Rhopalosiphum padi* . Insect Sci. 27, 69–85. doi: 10.1111/1744-7917.12606 29797656 PMC7379937

[B20] Leybourne D. J. Melloh P. Martin E. A. (2022). Common facultative endosymbionts do not influence sensitivity of cereal aphids to pyrethroids. Agr. Forest. Entomol., 25, 1–11. doi: 10.1111/afe.12539

[B21] Leybourne D. J. Valentine T. A. Bos J. I. B. Karley A. J. (2020b). A fitness cost resulting from hamiltonella defensa infection is associated with altered probing and feeding behaviour in *Rhopalosiphum padi* . J. Exp. Biol. 223, jeb207936. doi: 10.1242/jeb.207936 31822555

[B22] Li Q. Fan J. Sun J. Wang M. Chen J. (2018). Effect of the secondary symbiont *Hamiltonella defensa* on fitness and relative abundance of *Buchnera aphidicola* of wheat aphid, *Sitobion miscanthi* . Front. Microbiol. 9. doi: 10.3389/fmicb.2018.00582 PMC5884939 29651279

[B23] Li Q. Fan J. Sun J. Zhang Y. Hou M. Chen J. (2019). Anti-plant defense response strategies mediated by the secondary symbiont *Hamiltonella defensa* in the wheat aphid *Sitobion miscanthi* . Front. Microbiol. 10. doi: 10.3389/fmicb.2019.02419 PMC6823553 31708894

[B24] Li Q. Sun J. Qin Y. Fan J. Zhang Y. Tan X.. (2021). Reduced insecticide susceptibility of the wheat aphid *Sitobion miscanthi* after infection by the secondary bacterial symbiont *Hamiltonella defensa* . Pest Manage. Sci. 77, 1936–1944. doi: 10.1002/ps.6221 33300163

[B25] Liu X. Lei H. Chen F. (2019). Infection pattern and negative effects of a facultative endosymbiont on its insect host are environment-dependent. Sci. Rep. 9, 4013. doi: 10.1038/s41598-019-40607-5 30850675 PMC6408509

[B26] Łukasik P. Dawid M. A. Ferrari J. Godfray H. C. (2013). The diversity and fitness effects of infection with facultative endosymbionts in the grain aphid, *Sitobion avenae* . Oecologia 173, 985–996. doi: 10.1007/s00442-013-2660-5 23624672

[B27] Luo C. Gatti J.-L. Monticelli L. S. Poirié Marylène. Desneux N. Zhao H.. (2020a). An increased risk of parasitism mediated by the facultative symbiont *Regiella insecticola* . J. Pest Sci. 93, 737–745. doi: 10.1007/s10340-019-01189-3

[B28] Luo C. Monticelli L. S. Li D. Ahmed S. S. Pandharikar G. G. Zhao H.. (2020b). Comparison of life-history traits and resistance for *Sitobion avenae* (Fabricius) harboring a facultative symbiont. Entomol. Gen. 40, 39–47. doi: 10.1127/entomologia/2019/0823

[B29] Martinez A. J. Weldon S. R. Oliver K. M. (2014). Effects of parasitism on aphid nutritional and protective symbioses. Mol. Ecol. 23, 1594–1607. doi: 10.1111/mec.12550 24152321

[B30] Mathé-Hubert H. Kaech H. Ganesanandamoorthy P. Vorburger C. (2019). Evolutionary costs and benefits of infection with diverse strains of *Spiroplasma* in pea aphids. Evolution 73, 1466–1481. doi: 10.1111/evo.13740 30990223

[B31] McLean A. H. Godfray H. C. (2015). Evidence for specificity in symbiont-conferred protection against parasitoids. Proc. Biol. Sci. 282, 20150977. doi: 10.1098/rspb.2015.0977 26136451 PMC4528558

[B32] Meseguer A. S. Manzano-Marín A. Coeur d’Acier A. Clamens A.-L. Godefroid M. Jousselin E. (2017). *Buchnera* has changed flatmate but the repeated replacement of co-obligate symbionts is not associated with the ecological expansions of their aphid hosts. Mol. Ecol. 26, 2363–2378. doi: 10.1111/mec.13910 27862540

[B33] Montllor C. B. Maxmen A. Purcell A. H. (2002). Facultative bacterial endosymbionts benefit pea aphids *Acyrthosiphon pisum* under heat stress. Ecol. Entomol. 27, 189–195. doi: 10.1046/j.1365-2311.2002.00393.x

[B34] Moran N. A. McCutcheon J. P. Nakabachi A. (2008). Genomics and evolution of heritable bacterial symbionts. Annu. Rev. Genet. 42, 165–190. doi: 10.1146/annurev.genet.41.110306.130119 18983256

[B35] Mouton L. Henri H. Charif D. Boulétreau M. Vavre F. (2007). Interaction between host genotype and environmental conditions affects bacterial density in *Wolbachia* symbiosis. Biol. Lett. 3, 210–213. doi: 10.1098/rsbl.2006.0590 17251124 PMC2375926

[B36] Myint Y. Y. Bai S. Zhang T. Babendreier D. He K. Wang Z. (2021). Molecular and morphological identification of *Trichogramma* (Hymenoptera: Trichogrammatidae) species from Asian corn borer (Lepidoptera: Crambidae) in Myanmar. J. Econ. Entomol. 114, 40–49. doi: 10.1093/jee/toaa253 33558900

[B37] Neiers F. Saliou J. M. Briand L. Robichon A. (2021). Adaptive variation of *Buchnera* endosymbiont density in aphid host *Acyrthosiphon pisum* controlled by environmental conditions. ACS Omega 6, 17902–17914. doi: 10.1021/acsomega.1c01465 34308025 PMC8296009

[B38] Oliver K. M. Higashi C. H. (2019). Variations on a protective theme: *Hamiltonella defensa* infections in aphids variably impact parasitoid success. Curr. Opin. Insect Sci. 32, 1–7. doi: 10.1016/j.cois.2018.08.009 31113620

[B39] Oliver K. M. Smith A. H. Russell J. A. (2014). Defensive symbiosis in the real world - advancing ecological studies of heritable, protective bacteria in aphids and beyond. Funct. Ecol. 28, 341–355. doi: 10.1111/1365-2435.12133

[B40] Parker B. J. Spragg C. J. Altincicek B. Gerardo N. M. (2013). Symbiont-mediated protection against fungal pathogens in pea aphids: a role for pathogen specificity? Appl. Environ. Microbiol. 79, 2455–2458. doi: 10.1128/AEM.03193-12 23354709 PMC3623210

[B41] Patel V. Chevignon G. Manzano-Marín A. Brandt J. W. Strand M. R. Russell J. A.. (2019). Cultivation-assisted genome of candidatus *Fukatsuia* symbiotica; the enigmatic “X-type”symbiont aphids. Genome Biol. Evol. 11, 3510–3522. doi: 10.1093/gbe/evz252 31725149 PMC7145644

[B42] Perreau J. Zhang B. Maeda G. P. Kirkpatrick M. Moran N. A. (2021). Strong within-host selection in a maternally inherited obligate symbiont: *Buchnera* and aphids. Proc. Natl. Acad. Sci. U S A 118, e2102467118. doi: 10.1073/pnas.2102467118 34429360 PMC8536349

[B43] Pons I. Scieur N. Dhondt L. Renard M. E. Renoz F. Hance T. (2022). Pervasiveness of the symbiont *Serratia symbiotica* in the aphid natural environment: distribution, diversity and evolution at a multitrophic level. FEMS. Microbiol. Ecol. 98, 1–15. doi: 10.1093/femsec/fiac012 35142841

[B44] Scarborough C. L. Ferrari J. Godfray H. C. (2005). Aphid protected from pathogen by endosymbiont. Science 310, 1781. doi: 10.1126/science.1120180 16357252

[B45] Shigenobu S. Watanabe H. Hattori M. Sakaki Y. Ishikawa H. (2000). Genome sequence of the endocellular bacterial symbiont of aphids *Buchnera* sp. APS. Nature 407, 81–86. doi: 10.1038/35024074 10993077

[B46] Simon J. C. Boutin S. Tsuchida T. Koga R. Le Gallic J. F. Frantz A.. (2011). Facultative symbiont infections affect aphid reproduction. PLoS One 6, e21831. doi: 10.1371/journal.pone.0021831 21818272 PMC3144876

[B47] Simonet P. Duport G. Gaget K. Weiss-Gayet M. Colella S. Febvay G.. (2016). Direct flow cytometry measurements reveal a fine-tuning of symbiotic cell dynamics according to the host developmental needs in aphid symbiosis. Sci. Rep. 6, 19967. doi: 10.1038/srep19967 26822159 PMC4731799

[B48] Skaljac M. Kirfel P. Grotmann J. Vilcinskas A. (2018). Fitness costs of infection with *Serratia symbiotica* are associated with greater susceptibility to insecticides in the pea aphid *Acyrthosiphon pisum* . Pest Manage. Sci. 74, 1829–1836. doi: 10.1002/ps.4881 29443436

[B49] Tsuchida T. Koga R. Horikawa M. Tsunoda T. Maoka T. Matsumoto S.. (2010). Symbiotic bacterium modifies aphid body color. Science 330, 1102–1104. doi: 10.1126/science.1195463 21097935

[B50] Vogel K. J. Moran N. A. (2011). Effect of host genotype on symbiont titer in the aphid-*Buchnera* symbiosis. Insects 2, 423–434. doi: 10.3390/insects2030423 26467737 PMC4553553

[B51] Vorburger C. Gehrer L. Rodriguez P. (2010). A strain of the bacterial symbiont *Regiella insecticola* protects aphids against parasitoids. Biol. Lett. 6, 109–111. doi: 10.1098/rsbl.2009.0642 19776066 PMC2817266

[B52] Vorburger C. Rouchet R. (2016). Are aphid parasitoids locally adapted to the prevalence of defensive symbionts in their hosts? BMC Evol. Biol. 16, 271. doi: 10.1186/s12862-016-0811-0 27955622 PMC5153875

[B53] Wang D. Shi X. Dai P. Liu D. Dai X. Shang Z.. (2016). Comparison of fitness traits and their plasticity on multiple plants for *Sitobion avenae* infected and cured of a secondary endosymbiont. Sci. Rep. 6, 23177. doi: 10.1038/srep23177 26979151 PMC4793262

[B54] Whelan J. A. Russell N. B. Whelan M. A. (2003). A method for the absolute quantification of cDNA using real-time PCR. J. Immunol. Methods 278, 261–269. doi: 10.1016/s0022-1759(03)00223-0 12957413

[B55] Wilkinson T. L. Adams D. Minto L. B. Douglas A. E. (2001). The impact of host plant on the abundance and function of symbiotic bacteria in an aphid. J. Exp. Biol. 204, 3027–3038. doi: 10.1242/jeb.204.17.3027 11551991

[B56] Wilson A. C. C. Massonnet B. Simon J. C. Prunier-Leterme N. Dolatti L. Llewellyn K. S.. (2004). Cross-species amplification of microsatellite loci in aphids: assessment and application. Mol. Ecol. Notes 4, 104–109. doi: 10.1046/j.1471-8286.2004.00584.x

[B57] Wu T. P. Monnin D. Lee R. A. R. Henry L. M. (2022). Local adaptation to hosts and parasitoids shape *Hamiltonella defensa* genotypes across aphid species. Proc. Biol. Sci. 289, 20221269. doi: 10.1098/rspb.2022.1269 36285493 PMC9597410

[B58] Zhang B. Leonard S. P. Li Y. Moran. N. A. (2019). Obligate bacterial endosymbionts limit thermal tolerance of insect host species. Proc. Natl. Acad. Sci. U S A 116, 24712–24718. doi: 10.1073/pnas.1915307116 31740601 PMC6900525

[B59] Zhang Y. Cao W. Zhong L. Godfray H. C. J. Liu X. (2016). Host plant determines the population size of an obligate symbiont (*Buchnera aphidicola*) in aphids. Appl. Environ. Microbiol. 82, 2336–46. doi: 10.1128/AEM.04131-15 26850304 PMC4959500

[B60] Zytynska S. E. Tighiouart K. Frago E. (2021). Benefits and costs of hosting facultative symbionts in plant-sucking insects: A meta-analysis. Mol. Ecol. 30, 2483–2494. doi: 10.1111/mec.15897 33756029

