# Supplementary material for: Secondary symbionts affect aphid fitness and the titer of primary symbiont
Source: Front Plant Sci. 2023 Jan 27;14:1096750. doi: 10.3389/fpls.2023.1096750 (PMC9933779; doi:10.3389/fpls.2023.1096750)
Supplement: Supplementary file 1 [file Table_1.docx]

Supplementary Material

# Supplementary Table

Table S1 Primers used in this study.

| Application | Target genes | Primers | | Product (kb) | Tm (°C) | References |
| --- | --- | --- | --- | --- | --- | --- |
|  |  | Names | Sequences (5'-3') |  |  |  |
| Endosymbiont detection | S-symbionts | 10F  480R | AGTTTGATCATGGCTCAGATTG  CACGGTACTGGTTCACTATCGGTC | 2.0-2.5 | 56 | (Sandström et al., 2001) |
|  | *B. aphidicola* | Buch16S1F Buch16S1R | GAGCTTGCTCTCTTTGTCGGCAA  CTTCTGCGGGTAACGTCACGAA | 0.4 | 60 | (Sakurai et al., 2005) |
|  | *H. defensa* | T99F  1507R | AGTGAGCGCAGTTTACTGAG  TACCTTGTTACGACTTCACCCCAG | 1.39 | 56 | (Sandström et al., 2001) |
|  | *R. insecticola* | Reg-F  Reg-R | GTCTGGGGATCTGGCTTATG  CGTAGCATTCTGATCCACGA | 1.36 | 56 | In this study |
| *R. maidis* identification | *COⅠ* | LCO1490  HCO2198 | GGTCAACAAATCATAAAGATATTGG  TAAACTTCAGGGTGACCAAAAAATCA | 0.68 | 50 | (Guo et al., 2019) |
| Microsatellite loci PCR | *R3.171* | R3.171-F  R3.171-R | TGTACATCGTAAGACGTAAAACGAC  CAAAGCAATACCGCATAACG | - | 54 | (Leybourne et al., 2020) |
|  | *R5.10* | R5.10-F  R5.10-R | CCGACTAAGCTTAATATTGTTTG  CGGTTCGGAGAACATAAGAG | - | 50 | (Leybourne et al., 2020) |
|  | *S17b* | S17b-F  S17b-R | TTCTGGCTTCATTCCGGTCG  CGTCGCGTTAGTGAACCGTG | - | 59 | (Wilson et al., 2004) |
| qPCR | *B. aphidicola* | qBuch-F  qBuch-R | CACACTGGAACTGAGACACG  CTTCTTCATACACGCGGCAT | 0.1 | 60 | In this study |
|  | *H. defensa* | qHam-F  qHam-R | GGTCGCTAGAGTTTTCTAGAGGG  CCACAGTTCAAGACCGCAA | 0.18 | 60 | In this study |
|  | *R. insecticola* | qReg-F  qReg-R | GCGGTAATGGCGTACCTAGGCG  CTTACCGCCTACATGCCCTTTA | 0.33 | 60 | In this study |
|  | *Ef1α* | qEf1α-F  qEf1α-R | AGTCAGCAGTTACATCAAGAAGA  ACCATCAGCCTTTCCTTCTTT | 0.18 | 60 | In this study |

# References

Guo, J., Liu, X., Poncelet, N., He, K., Francis, F., and Wang, Z. (2019). Detection and geographic distribution of seven facultative endosymbionts in two *Rhopalosiphum* aphid species. *MicrobiologyOpen.* 8, e00817. doi: 10.1002/mbo3.817

Leybourne, D. J., Bos, J. I. B., Valentine, T. A., and Karley, A. J. (2020). The price of protection: a defensive endosymbiont impairs nymph growth in the bird cherry-oat aphid, *Rhopalosiphum padi*. *Insect Sci*. 27, 69-85. doi: 10.1111/1744-7917.12606.

Sandström, J. P., Russell, J. A., White, J. P., and Moran, N. A. (2001). Independent origins and horizontal transfer of bacterial symbionts of aphids. *Mol*. *Ecol*. 10, 217-228. doi.org/10.1046/j.1365-294X.2001.01189.x

Sakurai, M., Koga, R., Tsuchida, T., Meng, X., and Fukatsu, T. (2005). *Rickettsia* symbiont in the pea aphid *Acyrthosiphon pisum*: novel cellular tropism, effect on host fitness, and interaction with the essential symbiont *Buchnera*. *Appl. Environ. Microbiol.* 71, 4069-4075. doi: 10.1128/AEM.71.7.4069-4075.2005

Wilson, A. C. C., Massonnet, B., Simon, J-C., Prunier-Leterme, N., Dolatti, L., Llewellyn, K. S., et al. (2004). Cross-species amplification of microsatellite loci in aphids: Assessment and application. *Mol*. *Ecol*. *Notes*. 4,104-109. doi: 10.1046/j.1471-8286.2003.00584.x
